# Archaeal and Bacterial Content in a Two-Stage Anaerobic System for Efficient Energy Production from Agricultural Wastes

**DOI:** 10.3390/molecules27051512

**Published:** 2022-02-23

**Authors:** Lyudmila Kabaivanova, Venelin Hubenov, Lyudmila Dimitrova, Ivan Simeonov, Haoping Wang, Penka Petrova

**Affiliations:** 1 Institute of Microbiology, Bulgarian Academy of Sciences, 1113 Sofia, Bulgaria; vhubenov7@gmail.com (V.H.); lus22@abv.bg (L.D.); issim@abv.bg (I.S.); 2 French-Chinese Laboratory LaFCAS, School of Automation, Nanjing University of Science and Technology, Nanjing 210094, China; hp.wang@njust.edu.cn

**Keywords:** anaerobic digestion, wheat straw, two-stage performance, microbial consortia, metagenomics

## Abstract

Anaerobic digestion (AD) is a microbially-driven process enabling energy production. Microorganisms are the core of anaerobic digesters and play an important role in the succession of hydrolysis, acidogenesis, acetogenesis, and methanogenesis processes. The diversity of participating microbial communities can provide new information on digester performance for biomass valorization and biofuel production. In this study anaerobic systems were used, operating under mesophilic conditions that realized biodegradation processes of waste wheat straw pretreated with NaOH—a renewable source for hydrogen and methane production. These processes could be managed and optimized for hydrogen and methane separately but combining them in a two-stage system can lead to higher yields and a positive energy balance. The aim of the study was to depict a process of biohydrogen production from lignocellulosic waste followed by a second one leading to the production of biomethane. Archaeal and bacterial consortia in a two-stage system operating with wheat straw were identified for the first time and the role of the most important representatives was elucidated. The mixed cultures were identified by the molecular-biological methods of metagenomics. The results showed that biohydrogen generation is most probably due to the presence of *Proteiniphilum saccharofermentans*, which was 28.2% to 45.4% of the microbial community in the first and the second bioreactor, respectively. Archaeal representatives belonging to *Methanobacterium formicicum* (0.71% of the community), *Methanosarcina spelaei* (0.03%), *Methanothrix soehngenii* (0.012%), and *Methanobacterium beijingense* (0.01%) were proven in the methane-generating reactor. The correlation between substrate degradation and biogas accumulation was calculated, together with the profile of fatty acids as intermediates produced during the processes. The hydrogen concentration in the biogas reached 14.43%, and the Methane concentration was 69%. Calculations of the energy yield during the two-stage process showed 1195.89 kWh·t^−1^ compared to a 361.62 kWh·t^−1^ cumulative yield of energy carrier for a one-stage process.

## 1. Introduction

As the available oil reserves continue to be depleted, it is necessary to find alternative, sustainable energy sources that compensate for the growing energy demands worldwide. Although there are many different types of industries across the world, agriculture, together with the pulp and paper industries are to a great extent the major source of environmental pollution that produces large amounts of lignocellulosic wastes [1]. Lignocellulose is the main component of plant biomass and is, hence, ubiquitous. Lignocellulosic biomass has attracted great interest in recent years for energy production due to its renewability and carbon-neutral nature [2]. A more detailed knowledge of lignocellulosic waste utilization will facilitate managing this problem in the environment to reach sustainable development. Lignocellulosic substrates are not easy for biodegradation on account of their complex structure. Lignin can be partially removed by chemical or physical pretreatment, which could favor efficient bioconversion [3]. Effective pretreatment before anaerobic digestion could break down the linkage between polysaccharides and lignin and make cellulose and hemicelluloses more accessible to bacteria [4].

Anaerobic digestion (AD) of organic wastes is an attractive biotechnological alternative in the field of renewable energy source utilization with the aim of biofuel production. Generally, this process consists of liquefaction and hydrolysis of insoluble compounds and gasification of intermediates, which is accompanied by the partial or complete mineralization and humification of the organic substance [5,6]. Microbial cellulose utilization is responsible for one of the broadest material flows in the biosphere [7]. Waste treatment helps solve some ecological problems by reduction of their hazardous effects on the biosphere. Anaerobic digestion is a multi-step process leading to hydrogen production as an intermediate product and methane released that could be used as an energy carrier. 

A number of microorganisms are reported that have the capability to degrade and utilize cellulose and hemicellulose as carbon and energy sources [8,9]. For increasing the efficiency and stability of AD, many efforts on the exact identification of the microorganisms from a variety of microbial groups have been made, as such discoveries on the structure and diversity of participating microbial communities can provide new information on digester performance for biogas and energy production. Knowing the connections between operational conditions, process stability, and microbial community dynamics is essential to enhance AD process efficiency and management [10]. Consecutive cooperation of the population of microorganisms enables the synthesis of certain products that are then used by another group of bacteria [11] comprising the four groups of microorganisms: hydrolyzers, acidogenic microorganisms, acetogens, and methanogens [12]. Mixed cultures often present improved performance over corresponding monocultures, as microorganisms sometimes lack some key metabolic pathways, which may be supplemented by others [13]. The great variety of microbial groups are the core of the digesters [14], increasing the efficiency and stability of AD, so many efforts on elucidating the participating microorganisms, their role, and relations have been made in this field [15]. In the hydrolysis and acidogenesis process, there are about 50 kinds of bacteria, such as *Clostridium*, *Bacteroides*, *Bifidobacterium, Butyrivibrio, Proteobacteria, Pseudomonas, Bacillus, Streptococcus, Eubacterium,* and so on [16]. *Methanobacterium, Methanococcus, Methanobrevibacter, Methanomicrobium, Methanosarcina,* and *Methanosaeta* are the main microorganisms responsible for the methane production [17]. One key point is the requirement to find which one is the key component of the microbial community and which conditions are the most appropriate to achieve a high-efficiency process or lead to a failure process. Different groups of anaerobic microorganisms with specific growth and development conditions, physiological properties, and metabolic activities are involved [18]. Enhancement of the biodigestibility of lignocellulose by biological processes is a promising strategy because of its low capital cost, and low consumption of energy and chemicals. Symbiotic multi-species cellulose-degrading consortia, ranging from dual-species systems to complex microcosms, represent a good candidate for enhancing biomass degradation during biotechnological processes [19,20]. Although a defined consortium of microbes is desirable, the ecological complexity of consortia makes isolation of the required species difficult. Several methods have been applied to investigate the microbial diversity in the anaerobic digesters: a clone library of 16S rRNA genes, denaturing gradient gel electrophoresis (DGGE) analysis, fluorescence in situ hybridization (FISH), etc. Metagenomics is an efficient method for determining the complex microbiota structure and performing metabolic mechanism analysis. It is applied for elucidation of the community structure, and a metabolic pathway analysis can determine the mechanism of lignocellulosic substrate degradation [21,22].

This study aimed to analyze the processes of biohydrogen and biomethane production by wheat straw utilization with the participating microbial consortia therein to create a stable and effective two-stage process of anaerobic digestion, as by controlling and monitoring each of the microbiological, operational, and chemical parameters of AD, its performance may be enhanced.

## 2. Materials and Methods

### 2.1. Experimental Setup

Laboratory bioreactors, constructed in the Laboratory of Bioremediation and Biofuels (Institute of Microbiology, Bulgarian Academy of Sciences) with working volumes of 10 dm^3^ and 80 dm^3^ and 50 rpm stirrers were used. Both operated in a mesophilic mode (35–37 °C). Electronic regulators were used for measuring and controlling the temperature. The accuracy of the regulation under normal operation was ± 0.5 °C. Stirring was performed by constant electric motors and was about 100 rpm for both bioreactors. After each feeding, a purge was carried out to ensure an anaerobic environment. The substrate used was wheat straw pretreated with NaOH water solution that contained 4 g of NaOH for 100 g straw for 24 h at 55 °C [23]. Anaerobic cultivation conditions were provided using the corresponding techniques and appliances.

### 2.2. Inoculum Preparation

A working methane-generating anaerobic digester was used as a source of bacterial inoculum. This digester was operated at a mesophilic temperature in a fed-batch mode with native wheat straw as the sole substrate. As an inoculum for the hydrogen-producing process (in the first bioreactor), a pretreated digestate from the same methanogenic reactor was used. The preparation of the inoculum comprised a few steps: filtration, washing (saline solution), centrifugation (3000× *g* rpm), and, the most important, a thermal pretreatment at 80 °C for 15 min for the inactivation of methanogens. For the start-up of the methane fermentation process in the second bioreactor, digestate from the already-working methane tank was used. In all experiments, the inoculum was added in 10% (*v*/*v*) regarding the working volume of each bioreactor.

### 2.3. Analytical Methods

#### 2.3.1. Biogas Volume Measurement

The biogas volume was measured using a water displacement system, where the gas bubbled into the graduated measuring cylinder displaced the water from the cylinder into the container. The concentration of methane (CH_4_) was measured with an automatic gas analyzer “Dräger“, X-am 7000 (Lubeck, Germany). Carbon dioxide (CO_2_) and hydrogen (H_2_) were followed using a Gasboard gas analyzer (Wuhan, China, 3100D).

#### 2.3.2. Microscopic Observation

Microscopy examination and Gram staining were conducted under a Leika microscope (Wetzlar, Germany) with × 1000 magnification. All information gathered from the microscopy examinations was used to characterize the participating species. A digital camera was used for documentation of the microscopic images.

#### 2.3.3. Cellulose Concentration Estimation

The cellulose concentration was estimated by a spectrophotometer (JENWAY 6305, Dunmow, Essex, UK) using an anthrone reagent according to Updegraff [24].

#### 2.3.4. Volatile Fatty Acid Determination

Volatile fatty acid concentrations were measured by a Focus GC gas chromatograph (Thermo Scientific, Waltham, MA, USA), equipped with a split/splitless injector, TG-WAXMS (length: 30 m, ID: 0.25 mm, film: 0.25 μm) column, and FID (Zurich, Switzerland).

### 2.4. Metagenome Sequencing and Bioinformatics Analysis

The metagenome library construction and sequencing were conducted by Macrogen Inc. (Seoul, Korea). For library construction, total DNA was extracted from a sample using a GeneJET Genomic DNA Purification Kit (Thermo Scientific™, Waltham, MA, USA). The preparation of the 16S metagenomic sequencing library for bacteria was performed using a primer pair that targeted the V3-V4 region (Macrogen primer set), while the archaeal metagenomic library was constructed using the primers 519F_Arch 5′CAGCMGCCGCGGTAA3′ and 806R_Arch 5′GGACTACVSGGGTATCTAAT3′ [25]. Both libraries were created the with Herculase II Fusion DNA Polymerase Nextera XT Index Kit V2. The sequencing (Illumina platform) was conducted with a reading length of 301 bp and FastQC quality control. The total reads were 5,402,112 bases. The percentage of Q20 quality reads was 91.02%.

### 2.5. Statistical Analysis

All statistical analyses were performed using Microsoft Excel software. The data are reported as means ± SD. A *p*-value lower than 0.05 was considered statistically significant.

## 3. Results and Discussion

Microbial fermentation processes in the biosphere are responsible for the greater part of the biologically produced hydrogen. Biohydrogen is defined as hydrogen produced by bacteria, archaea, or algae from cultivations and waste organic materials [26]. These organisms decompose organic matter into carbon dioxide and hydrogen. The microbiome residing in anaerobic digesters drives the anaerobic digestion process. All strains of methanogenic bacteria utilize H_2_ as an electron donor for methanogenesis and growth. Otherwise, various complex substrates could not be converted to biogas as a renewable source of energy. We have analyzed the process of two-stage anaerobic digestion enabled by the complex communities of microorganisms present in both AD bioreactors. Using this approach, we tried to obtain new information on the composition and diversity of the biogas-producing microbial consortia with the aim to improve AD efficiency and stability.

### 3.1. Biogas Production Using Cascade Bioreactors

**Figure 1:**
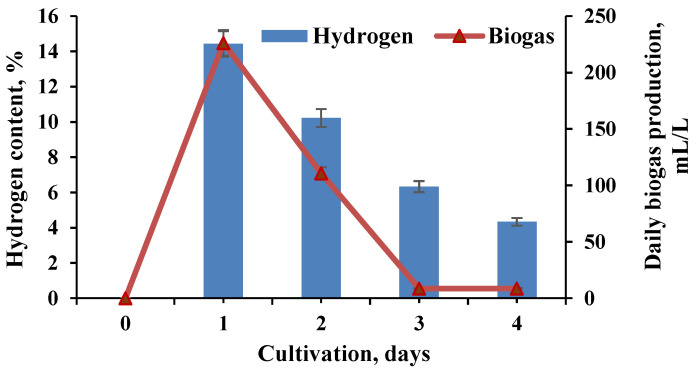
Dynamics of biogas (biohydrogen) production in the first BR of the cascade.

NaOH pretreated wheat straw with a loading of 10 g/L was the substrate subjected to anaerobic digestion in the system of two bioreactors (BR). The experiments were carried out in a semi-continuous mode. The released gas volume and hydrogen therein are presented in Figure 1.

**Figure 2:**
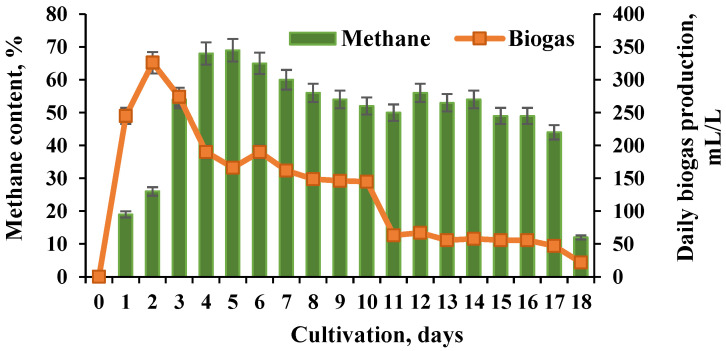
Biomethane production in BR2 of the cascade.

The pH was maintained in the range of 4.9 to 5.5 and controlled during the whole process. The hydrogen concentration in the biogas reached 14.43%. The yields of biogas were also measured in the second BR of the cascade and were estimated after the addition of 170 mL of liquid from the hydrogen-generating BR from the process, operating with wheat straw pretreated with NaOH (BR1). The maximum content of methane reached 69% (Figure 2).

**Figure 3:**
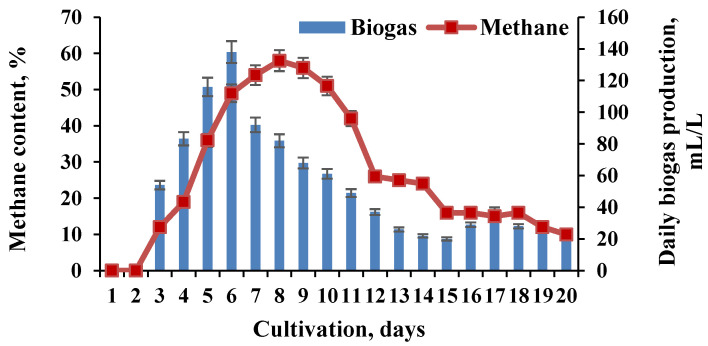
Biomethane production in a single methanogenic BR.

The pH in the second BR was maintained in the range of 5.5 to 7.0 during the whole process. For comparison, performing the process with the same substrate, pretreatment, and loading in a single-stage system realized the highest methane value of 58% (Figure 3).

Involving a two-stage AD system, where the processes were divided into a cascade of two separate bioreactors, the division of the processes into two consecutive bioreactors lead to higher energy yields for the two-stage system, realizing higher hydrogen and methane production compared to the traditional single-stage methanogenic process with different types of substrates as mentioned in the literature [27,28]. The calculations for the energy balance in the system lead to the conclusion that it is three-fold in favor of the two-stage process (Table 1).

The primary energy obtained during the process from untreated and pretreated biomass (wheat straw), which was expressed as kWh·t^−1^ raw material, was calculated by Equation (1):

Ee = Y. LHV. (mp/mu)(1)

where Y is the yield of the respective energy carrier in m^3^·t^−1^, LHV (lower heating value) is assumed to be 9.94 kWh·Nm^−3^ (for methane) and 2.99 kWh·Nm^−3^ (for hydrogen), and mu and mp are the sample masses before and after pretreatment. According to our results and calculations, the energy yield in a two-stage system is 3.3-fold higher than the single-stage process. The biogas production by the two-stage AD process enabled 18.5% higher energy recovery than single-stage AD when treating high-moisture municipal solid waste [29].

Fermentation is one of the most promising methods of biohydrogen production from industrial wastewater, owing to its ease of operation and rapid hydrogen production, where a group of bacteria mainly carry out dark fermentation of industrial wastewater by the help of multienzyme systems to convert organics into hydrogen without oxygen [30].

According to Hosseinzadeh et al., [31], regarding H_2_ production efficiency, the combined dark fermentation with microbial electrolysis cell process was the best process in relation to large-scale performance.

**Figure 4:**
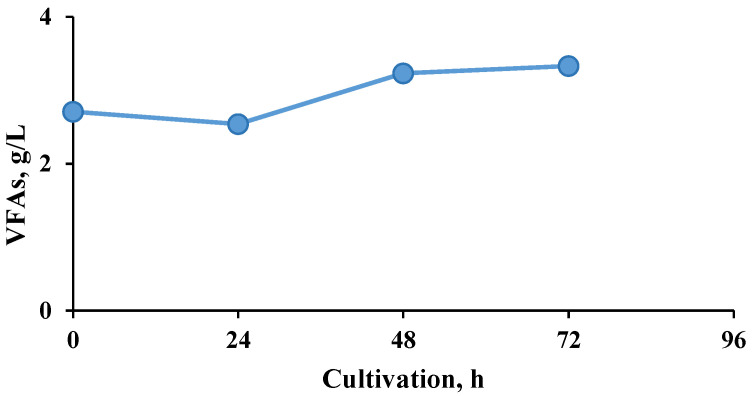
Dynamics of the total volatile fatty acid (VFA) concentration during the hydrogen production process.

**Figure 5:**
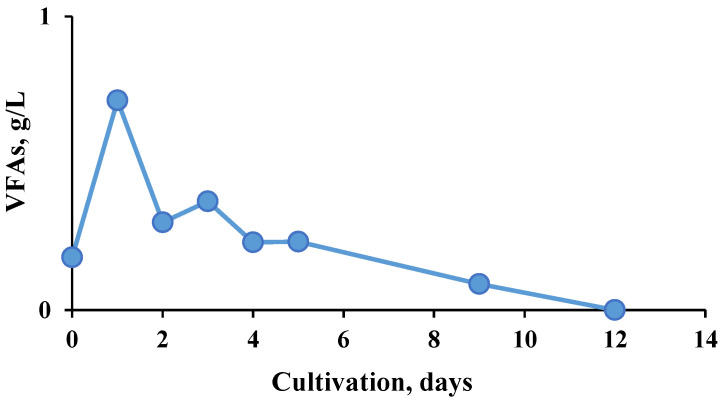
Dynamics of the total VFA concentration during the methanogenic process.

A prerequisite for higher methane production in the two-stage process was due to improved substrate hydrolysis, with increased amounts of volatile fatty acids (VFA) produced in the first bioreactor that were readily available in the second stage (Figure 4 and Figure 5). 

**Figure 6:**
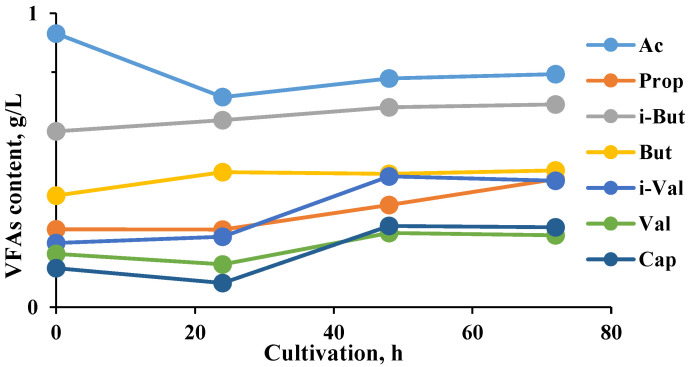
Profile of the volatile fatty acids (VFA) during the hydrogen generation process. Designations: Ac, acetate; Prop, propionate; i-But, isobutyrate; But, butyrate; i-Val, isovalerate; Val, valerate; Cap, capric acid.

**Figure 7:**
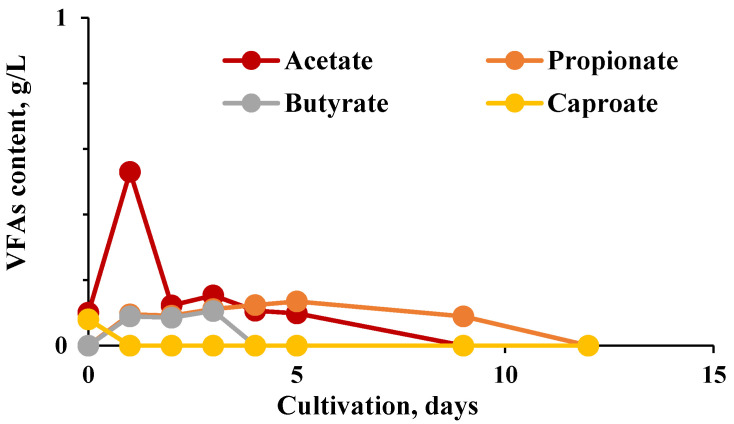
Profile of VFA during a methane-generating process. The values for i-But, i-Val, and Val are zero.

The consortia of microorganisms during biodegradation produced the following cocktail of VFA (Figure 6 and Figure 7).

The major volatile fatty acids produced during fermentation were essentially acetate and butyrate, suggesting that the typical butyrate-type fermentation could be achieved by the participating microbial consortia. Similar results were obtained using a different substrate, a mix of fruits and vegetables, as a carbon source by Likata et al., 2011 [32].

The main pathway of acidogenesis is through acetate, carbon dioxide, and hydrogen. An accumulation of lactate, ethanol, propionate, butyrate, and higher volatile fatty acids is the response of the bacteria to an increased hydrogen concentration in the medium [33]. In the absence of methanogens to utilize these substrates, hydrogen supports the overall degradation process, and organic acids accumulate, causing a decrease in pH, which could inhibit fermentation unless controlled. The overall performance of the anaerobic digestion system is affected by the concentration and proportion of the individual volatile fatty acids formed during the acidogenic stage because acetic and butyric acids are the preferred precursors for methane production [34].

### 3.2. Microbial Communities in the Two-Stage Anaerobic System Identified by Metagenomics

In many studies, the microbial community analysis provided crucial information to understand the anaerobic digestion process, which may help to improve its efficiency [35,36,37]. The main acidogenic and methanogenic species in the community of microorganisms in AD differ in the optimal conditions for their growth and development. Therefore, in AD in a single bioreactor (BR) with a one-stage process, the optimal conditions are selected, taking into account the slow-growing methanogens at the expense of the fast-growing acidogens, which affects the process efficiency.

**Figure 8:**
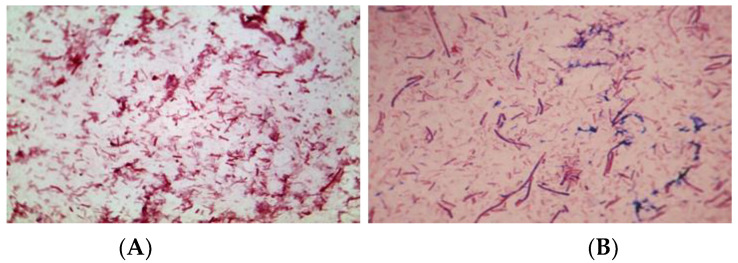
Gram-stained bacterial consortia under the microscopic view, (Leica, DMC4500 Digital Microscope Camera, magnification 1000×): (**A**) the consortium in the hydrogenic reactor; (**B**) the consortium in the methanogenic reactor.

Figure 8 reveals the microbes inhabiting the system of BRs by light microscopy. While in the first bioreactor the short rods and coccoid forms predominate, in the second BR, longer rod-shaped clostridial forms appeared. Indeed, the participating microbial community had a complex structure and probably used synergetic mechanisms in utilizing the wheat straw.

**Figure 9:**
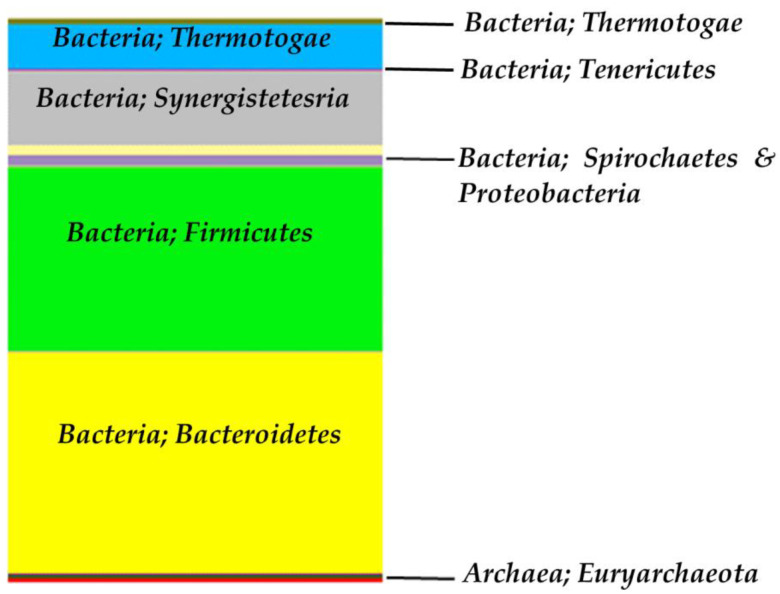
Biodiversity in the two-stage reactor in the anaerobic digestion system (the main phyla).

Figure 9 presents the main phyla in the anaerobic digestion system. It is evident, that bacteria are predominant in both bioreactors, while Archaea representatives are present in minor amount.

#### 3.2.1. Bacterial Biodiversity

The microbial community structures in different research works vary greatly, as they use different inocula, substrates, and conditions in the implementation of anaerobic biodegradation of lignocellulosic substrates. In this study, we observed that the first BR (with the main target product hydrogen) contained mainly the representatives of the genera Proteiniphilum, Prevotella, and Clostridium, followed by Caproiciproducens, Dechlorosoma, and Caloramator. The microbial community of our second fermenter (with the main target product methane) was dominated by the genera Proteiniphilum, Bacteroides, Anaerotaenia, Ruminiclostridium, and Hungateiclostridium. They had the potential to produce methane by the acetoclastic or hydrogenotrophic metabolic pathway.

**Figure 10:**
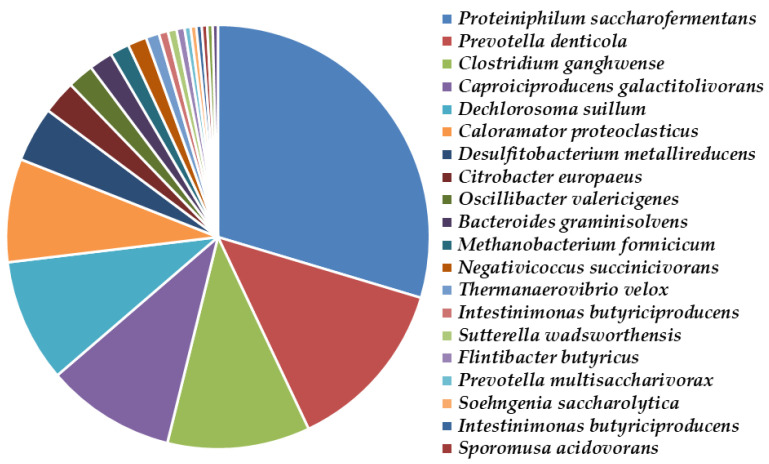
Species diversity in the hydrogen-producing microbial consortium inhabiting the first bioreactor.

**Figure 11:**
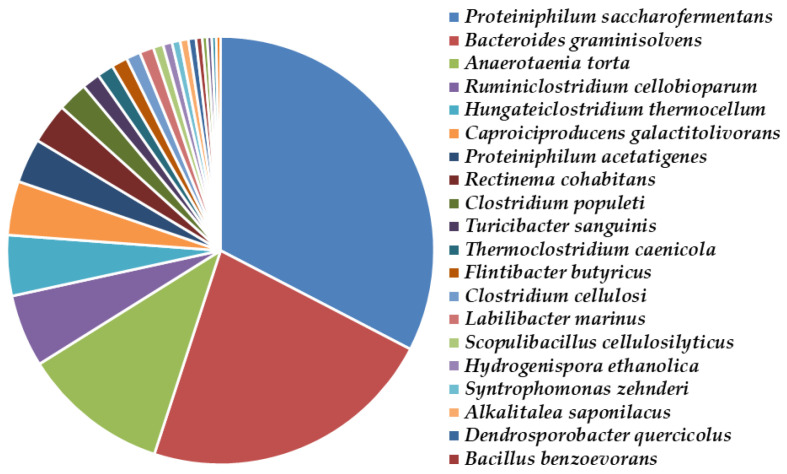
Species diversity in the methane-producing microbial consortium inhabiting the second bioreactor.

*Proteiniphilum saccharofermentans* comprised 28.2% to 45.4% of the microbial community in the first and the second bioreactors, respectively (Figure 10 and Figure 11).

The isolation of this species from an anaerobic mesophilic two-phase biogas reactor (fed with 95% maize silage and 5% wheat straw) was reported by Hanke et al. [38]. The strain *P. saccharofermentans* M3/6T produced acetate, propionate, and iso-valerate as volatile fatty acids, and carbon dioxide, hydrogen, acetate, formate, propionate, and iso-valerate as end-products of the fermentation process, suggesting that its function in the biogas production process is associated with the acidogenic phase [39]. It is known that *P. saccharofermentans* produces extracellular enzymes involved in the degradation of complex carbohydrates (β-glucan, xylan, arabinoxylan, starch, arabinogalactan, phosphoric acid-swollen cellulose (PASC), and carboxymethyl cellulose). According to the NCBI database, the *P. saccharofermentans* strain M3/6T contains various genes encoding carbohydrate-active enzymes, which are involved in the decomposition of pectin, arabinogalactan, hemicellulose (arabinan, xylan, mannan, and β-glucans), cellulose, starch, fructan, chitin, and pullulan [39]. Strain M3/6T is also able to degrade xylose, lyxose, mannose, and melibiose, as well as some amino acids (e.g., proline, alanine, and asparagine) to pyruvate [40]. The majority of the metabolites are converted to pyruvate [40]. All these abilities indicate that the species *P. saccharofermentans* increases biogas production.

The reductive acetogen *Proteiniphilum acetatigenes* was detected only in the methane-producing consortium and comprised 4.7% of the bacterial community (Figure 10). This species produces acetic acid from yeast extract, peptone, pyruvate, and L-arginine fermentation, as well as propionic acid and is known to increase methane production [41,42]. Interestingly, it has the potential to improve milk yield and milk fat in lactating cows by lowering somatic cell counts [43].

*Prevotella denticola* shared 12.7% of the bacterial consortium producing hydrogen (Figure 9). This species is Gram-negative, obligately anaerobic, rod-shaped, and belongs to the phylum *Bacteroides*. It has previously been isolated during biohydrogen production [44]. However, it was 0.3% of the community with methanogenic activity, similar to the observations of Mariakakis et al. [45]. The end products of *Prevotella denticola* metabolism during sucrose fermentation are mainly succinic and acetic acids, while smaller quantities of lactic acid can also be detected. Hydrogen is produced in the first case (hydrolysis of sucrose to succinic acid) and it is released when the metabolic pathway is directed to acetate production [45]. *Pr. denticola* cannot produce hydrogen. Conversely, these bacteria decrease the hydrogen yield due to competition with the hydrogen-producing organisms. Therefore, in order to improve the quality of biohydrogen production, the growth of *Pr. denticola* should be inhibited [46].

*Bacteroides graminisolvens* presented 31.2% of the bacterial community in the methane-producing BR (Figure 11). It was previously isolated from rice-straw residues in a mesophilic methanogenic reactor treating waste from cattle farms [47] and methanogenic sludge [48]. Bacteria from the genus *Bacteroides* were found to be predominant in enriched cultures with cellulose and hemicellulose as the sole carbon source. This species utilizes cellobiose, arabinose, xylose, fructose, galactose, glucose, mannose, lactose, maltose, melibiose, sucrose, raffinose, xylooligosaccharides, dextrin, glycogen, starch, pectin, xylan (birch wood), amygdalin, and salicin but cannot degrade carboxymethylcellulose and cellulose [47]. That is why their presence is awaited. According to Sarkar et al. [49], this species produces propionic and acetic acids.

Another predominant species in the methane-producing consortium was *Anaerotaenia torta* (4180 16S rDNA copies, or 15.4%), while in the hydrogen-BR-generated population its growth was inhibited (to only 0.05%). It was previously isolated from a methanogenic reactor of cattle waste from farms in Japan. The data show that this species utilizes the carbohydrates arabinose, ribose, xylose, fructose, galactose, glucose, mannose, rhamnose, cellobiose, xylan, and mannitol to generate acetate, ethanol, H_2_, and CO_2_ as end products [50]. Therefore, we assumed that this species is irrelevant to hydrogen production but grows in synergy with methane-producing bacteria.

*Clostridium ganghwense* was the third most abundant species in the first bioreactor (10.3%). There is a lack of information about *C. ganghwense*. This bacterial species was previously isolated from tidal flat sediment. It is known that this species produces glycerol, ethanol, and CO_2_ as end products of glucose fermentation [51].

*Caproiciproducens galactitolivorans* presented in both the hydrogen- and methane-generating consortia, as it was 9.4% of the first community and 5.7% of the second. This species was previously isolated from a wastewater treatment plant and was capable of producing caproic acid from galactitol [52], as well as acetate and butyrate [53]. According to Qin et al. [54], the representatives of the genus *Caproiciproducens* are the key hydrogen-producing bacteria when *Ruminiclostridium* spp. is missing, as in the case with our hydrogen-producing consortium. The *Caproiciproducens* spp. and the predominant cellulolytic species *Ruminiclostridium* spp. are usually in synergy in methanogenic bioreactors [55,56]. In the methane-generated population, *Ruminiclostridium cellobioparum* was 7.6% of the community. However, information about this species is scarce. It is known that it was isolated quite recently as part of the bacterial consortium from a lab-scale biogas fermenter fed with maize silage [57].

Information on the role of *Dechlorosoma suillum* in the two-stage process is scanty. It is known that this species dominates in environmental samples. According to Goud et al. [58], this strain was predominant in a control reactor for long-term fermentative biohydrogen production and has the ability to grow by a dissimilatory reduction of perchlorate and chlorate. This hypothesis coincides with our results: *Dechlorosoma suillum* was ranked as the fifth most abundant species (8.9%) in the first BR. In the second process, where the amount of methane was high, the species was very poorly represented (0.14%).

*Caloramator proteoclasticus* predominated in the hydrogen-producing consortium (2363 16S rDNA copies, 7.5%), while in the methane-generating population it was 0.07%. This species was previously isolated from methanogenic sludge and was known to ferment proteins and carbohydrates to acetic, formic, and lactic acids, ethanol, branched-chain fatty acids, and hydrogen [59]. Unfortunately, the high cell amount leads to a low hydrogen production rate [60], which makes it useless for hydrogen production [61]. 

*Hungateiclostridium thermocellum* presented with 1724 16S rDNA copies (6.4% of the methanogenic community, Figure 11). This species was isolated from a laboratory-scale biogas fermenter during the conversion of plant biomass to methane-rich biogas [62,63]. *H. thermocellum* is related to the degradation of cellulose and the production of methane, as was confirmed in our study.

The presence of *Desulfitobacterium metallireducens* was expressed in 1263 16S rDNA copies (4% of the hydrogen-forming community, Figure 10). These bacteria are capable of reducing metals and humic acids, as well as chlorinated compounds. It uses lactate, formate, and butyrate as electron donors for respiration and uses Fe (III), anthraquinone-2,6-disulfonate, and 3-chloro-4-hydroxyphenylacetate as electron acceptors [64]. Karatas et al. [65] have studied the species diversity in a hydrogen-based membrane biofilm reactor and found that the same bacteria were in a very high amounts, similar to our results.

For the first time, we proved the presence of *Rectinema cohabitans* in the methane-generating consortium. It was present with 1153 16S rDNA copies (4.3% of the methanogenic community, Figure 11). The only information about this bacterial strain is that it is capable of degrading naphthalene, reducing sulfate, and fermenting various sugars, such as D-glucose, D-fructose, lactose, and sucrose [66]. The species identification using molecular methods, such as metagenomics, revealed the microbial population involved in both bioreactors. Precise microbiome identification by molecular biology techniques overcame cultivation-based methods and allowed the identification of unculturable microorganisms, revealing the high diversity of microorganisms involved in AD [67].

#### 3.2.2. Archaeal Biodiversity

**Figure 12:**
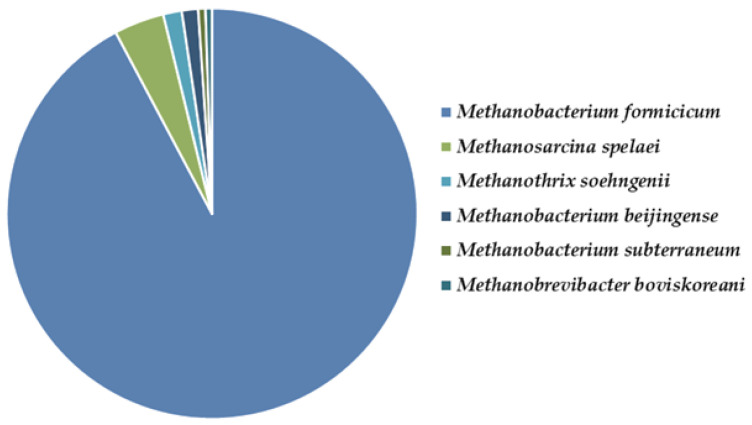
Archaeal biodiversity in the methane-producing microbial consortium. The ratio between archaeal species is presented.

As many of the genera belonging to Archaea are methanogenic, the archaeal biodiversity in the methanogenic bioreactor (BR2) was studied. Archaeal species were found to account for only 0.86% of the taxonomic diversity in the methanogenic bioreactor, while 99.14% of the specific sequences belonged to bacteria. In the sample were found the representatives of phylum *Euryarchaeota*, classes *Methanobacteria* and *Methanomicrobia* (Figure 12). 

Among them, the species *Methanobacterium formicicum* was the most abundant, comprising 0.71% of the community, followed by *Methanosarcina spelaei* (0.03%), *Methanothrix soehngenii* (0.012%), and *Methanobacterium beijingense* (0.010%). The other two archaeal species, *Methanobacterium subterraneum*, and *Methanobrevibacter boviskoreani,* were even less presented and were <0.005% of the methanogenic consortium.

Although it was known as an archaeal species often found in caw’s rumen, *Methanobacterium formicicum* was also studied in terms of methane bioproduction. The strain JF-1 was cultured with formate as the sole energy source in a pH-stat fermenter by Schauer and Ferry [68]. During the exponential phase, the methane production and formate consumption were linear functions of the growth rate. Hydrogen was produced in only trace amounts.

Ziganshin et al. [69] reported that the specific biogas production significantly correlates with Methanosarcinaceae presence, and *Methanosarcina spelaei*, thus conforming to our observation. *Methanobacterium beijingense* is known as a common representative in anaerobic digesters and is a part of the microbial flora, which plays an important role in the anaerobic degradation of organic compounds [70].

## 4. Conclusions

The two processes for hydrogen and methane production were combined and optimized for each biological step in the process of anaerobic digestion, which makes the whole system more stable and efficient. The most important advantage of a two-phase anaerobic digestion system consists in the possibility of producing hydrogen during the first acidogenic phase and subsequently producing methane during the second methanogenic phase while utilizing a waste wheat straw. The synergistic behavior of the newly identified various anaerobic microorganisms, which differ in the two separate BRs operating with wheat straw, results in the decomposition of complex organic renewable sources for obtaining the renewable energy carriers hydrogen and methane. Regarding the potential environmental impact, together with the economic aspect, it was proven that the energy released in a single-stage process is approximately 361.62 kWh·t^−1^, while 1195.89 kWh·t^−1^ were obtained for the two-stage process with solely wheat straw as a substrate. The microbiomes residing in the anaerobic digesters that target hydrogen and methane production were identified using metagenomics. The wide spectrum of defined microbial consortia could further be involved in effective processes for renewable energy production.

## Figures and Tables

**Table 1:** Energy yield for the single-stage and two-stage anaerobic digestion systems.

System	Energy Carrier	Cumulative Yield (cm^3^)	Yp *(m^3^·t^−1^)	LHV * (kWh·Nm^−3^)	Total Energy (kWh·t^−1^)	Total Energy (per System) (kWh·t^−1^)
Single-phase	Methane	29104.0	36.38	9.94	361.62	361.62
Two-phase	Hydrogen	134.7	4.49	2.99	13.43	1195.89
	Methane	95168.0	118.96	9.94	1182.46	

* Yp, the yield of energy carrier per ton of wheat straw added; LHV, lower heating value.

## Data Availability

Not applicable.

## Abbreviations

AD	anaerobic digestion
DGGE	denaturing gradient gel electrophoresis
FISH	fluorescence in situ hybridization
BR	bioreactor
Yp	Yield of energy carrier per ton wheat straw added, [m^3^·t^−1^]
LHV	lower heating value, [kWh·Nm^−3^]
Ee	primary energy obtained during the anaerobic digestion process, [kWh·t^−1^]
VFA	volatile fatty acids, [g/L]
Ac	acetate, [g/L]
Prop	propionate, [g/L]
i-But	isobutyrate, [g/L]
But	butyrate, [g/L]
i-Val	isovalerate, [g/L]
Val	valerate, [g/L]
Cap	capric acid, [g/L]
PASC	phosphoric acid-swollen cellulose
NCBI	National Center for Biotechnology Information
rDNA	ribosomal deoxyribonucleic acid
FID	flame ionization detector
SD	standard deviation
